# Increasing lactose concentration is a strategy to improve the proliferation of *Lactobacillus* *helveticus* in milk

**DOI:** 10.1002/fsn3.2076

**Published:** 2020-12-19

**Authors:** Jingyi Zang, Tao Wang, Dziugan Piotr, Hongfei Zhao, Bolin Zhang

**Affiliations:** ^1^ College of Biological Science & Biotechnology Beijing Key Laboratory of Forest Food Processing and Safety Beijing Forestry University Beijing China; ^2^ Institute of Fermentation Technology & Microbiology Lodz University of Technology Lodz Poland

**Keywords:** growth and metabolism, high‐cell‐density culture, *Lactobacillushelveticus*, lactose, proliferation

## Abstract

The aim of the research was to explore the effect of carbon sources on the proliferation of *Lactobacillus helveticus*. In this study, lactose was added to skim milk medium and the effects of carbon sources on the growth and proliferation of *Lactobacillus helveticus* in low‐ and high‐sugar media were compared from the aspects of metabolism‐related enzyme activity, proteomics, and transcriptomics. The results showed that under high‐sugar conditions, the rate of substance transport to cells and the Embden–Meyerhof–Parnas (EMP) pathway were significantly accelerated. The synthesis and metabolism of cells were significantly enhanced, which was beneficial to the rapid proliferation of cells. By increasing the lactose concentration in the medium and optimizing the culture method, the cell density of *Lactobacillus helveticus* reached 3.98 × 10^9^ CFU/ml; a good proliferation effect was obtained.

## Graphical abstract

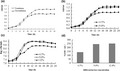

## 1 INTRODUCTION

Lactic acid bacteria (LAB) are the second most important microorganisms in the food industry. At present, there are many studies on LAB. *Lactobacillus helveticus* was added to the process of yogurt production to improve the fermentation and storage of yogurt (Zhou et al., 2019). In addition, *L. helveticus* was added to cheese ripening to facilitate the release of bioactive peptides (Baptista et al., 2018). *L. helveticus* has a strong proteolytic activity and a high content of peptides in fermented dairy products. Therefore, *L. helveticus* has the potential to produce bioactive peptides (Zhao et al., 2014). Commercial *L. helveticus* also has proteolytic activity (Naoki et al., 2020). Therefore, much attention has been focused on *L. helveticus* in China recently (Zhang et al., 2013).

Many researchers promoted the growth of LAB by analyzing the nutritional requirements of LAB and optimizing the nutrient composition of the medium. After skim milk was hydrolyzed by protease, proliferative factors such as peptone, yeast extract, and lactose were added to the medium to promote the proliferation of *Lactobacillus* (Li et al., 2007). Lactose, yeast extract, wort, and trisodium citrate were added to skim milk medium to promote the proliferation of *L. helveticus* (Wu et al., 2008). Whey protein hydrolysates were added to skim milk to promote the growth of *Lactobacillus acidophilus* and *Bifidobacterium* (Mccomas & Gilliland, 2003). There are studies on the proliferation of *Lactobacillus* with soy protein hydrolysates; soybean protein hydrolysate can promote the growth of *Streptococcus thermophilus* and *Lactobacillus bulgaricus* (Zhao, 2013). Yao added vitamins to skim milk medium to promote the growth of the strain. The results indicated that vitamin C, biotin, and vitamin B5 promote the proliferation of *L. helveticus* but have no significant effect on *Lactobacillus bulgaricus* (Yao, 2018). At present, in the studies promoting proliferation by regulating growth factors, the additives mainly included peptone, tryptone, soybean peptone and its hydrolysates, and vitamins. Adequate sources of nitrogen in milk had been demonstrated. However, there were few reports on the effects of carbon sources on the growth of *L. helveticus*. Maryam discovered that lactose–glucose was used as a complex carbon source to multiply *Lactobacillus plantarum*. The highest concentration of conjugated linoleic acid can be obtained. This indirectly explains the promoting effect of carbon sources on the growth of lactic acid bacteria (Maryam et al., 2020). The objectives of this study were to: (1) analyze the effect of lactose on the growth of *L. helveticus*; (2) explore the mechanism of the effect of lactose on the growth and metabolism of *L. helveticus*; (3) establish a high‐density culture system of *L. helveticus*.

## 2 MATERIALS AND METHODS

### 2.1 Strains and growth medium

*Lactobacillus helveticus* CICC 22171 was isolated from traditional Chinese cheese. Currently, it is preserved in the China industrial microbial culture preservation management center (CICC). The freeze‐dried strain was first inoculated in 12% skim milk medium. The media used in this study was De Man, Rogosa and Sharpe medium (MRS), low‐sugar medium, and high‐sugar medium. Low‐sugar medium consisted of 12.0% skimmed milk (3.2% protein, 4.5% lactose) and 5% yeast extract. The lactose content in the high‐sugar medium was 8.0%.

### 2.2 Effects of lactose on the growth of *L. helveticus*

To investigate the effect of lactose on *L. helveticus*, low‐sugar and high‐sugar media were selected for fermentation. The fermentation process was performed in a biological fermentation tank (BIOTECH‐3BG‐5BG, Baoxing Biological Equipment Co., Ltd., Shanghai, China) and the inoculation amount was 5%. The fermentation parameters were controlled as follows: 37°C, 85 rpm, and pH 6.0 (adjusted with 7.5 mol/L NaOH). During fermentation, samples were taken every hour to determine the cell density and viable count. For the determination of cell density, the collected fermentation broth was diluted with sterile distilled water in a ratio of 1:3. Cells were harvested by centrifugation (4°C, 8,000 × *g*, 10 min), and then washed twice with an equal volume of sterile saline. After that, the precipitate was collected and suspended in an equal volume of sterile saline and the absorbance was measured at a wavelength of 540 nm. Viable cells were counted by plating on MRS agar and incubating anaerobically at 37°C for 48 hr.

### 2.3 Mechanism of the effect of lactose on the growth and metabolism of *Lactobacillus helveticus*

#### 2.3.1 Effects of lactose on metabolism‐related enzyme activity and gene expression

*Lactobacillus helveticus* was fermented in low‐ and high‐sugar media. A Bradford protein concentration assay kit (Biyuntian Institute of Biotechnology, Jiangsu, China) was used to determine the protein concentration in bacterial cell extracts.

Activities of β‐galactosidase (β‐GAL), hexokinase (HK), 6‐phosphofructokinase (PFK), pyruvate kinase (PK), and lactate dehydrogenase (LDH) were measured using the corresponding kit (Keming Institute of Biotechnology, Jiangsu, China).

Gene expression of HK, PFK, PK, LDH, and β‐GAL in cDNA samples was determined by fluorescence quantitative PCR, with the 16S rRNA gene as the internal reference.

#### 2.3.2 Effect of lactose on protein expression

Total protein was extracted using a proteomics‐level bacterial protein extraction kit (786–258, Sangon Biotech Shanghai Co., Ltd., Shanghai, China). Total protein concentration was determined using a non‐interfering protein concentration assay kit (SK3071, Sangon Biotech Shanghai Co., Ltd., Shanghai, China). Differential protein expression was determined by two‐dimensional electrophoresis. Then, Image Master 2D Platinum (Version 7.0) was used for two‐dimensional electrophoresis analysis. MALDI‐TOF‐TOF‐MS analysis was carried out after the intragamentum extraction and enzymolysis.

#### 2.3.3 Analysis of the effect of lactose on gene expression of *L. helveticus* cells

A transcriptome method was used to investigate the effect of lactose on gene expression. The TRIzol kit (Sangon Biotech Shanghai Co., Ltd., Shanghai, China) was employed to extract total RNA. Total RNA from LAB was purified away from the 16S and 23S ribosomal RNA using the Ribo‐Zero Magnetic Kit (MRZB12424, Epicentre Biotechnologies, Wisconsin, USA); the rest of the sample was the bacterial mRNA. The transcriptome was sequenced using the Illumina HiSeq 2000 high‐throughput sequencing platform (Illumina, California, USA).

RNA sequencing bioinformatics analysis was performed to sequence the mRNA of the *L. helveticus* transcriptome. Sample data were obtained by single‐end sequencing on the HiSeq 2000. DEGseq was used for differential expression analysis. The screening conditions for differential genes were *p* < .05 and an absolute value of log 2 (fold change) that was greater than or equal to 1.

Gene Ontology (GO) enrichment analysis was performed to investigate the corresponding biological activities of differential genes. The Clusters of Orthologous Groups of proteins (COG) database was used for orthologous classification of gene products (proteins). Using the COGNITOR program, identified proteins were aligned with all proteins in the COG database and placed into the appropriate COG cluster. The Kyoto Encyclopedia of Genes and Genomes (KEGG) database was used to identify enzymatic pathways and biochemical substances.

### 2.4 Establish a high‐density culture system of *L. helveticus*

#### 2.4.1 Optimization of strain culture conditions

Skim milk medium (100 ml) was added to a 300 ml flask, and the cells were inoculated at 2%, 5%, 8%, and 10% of the total volume. Strains were incubated at 37°C for 10 hr. The viable count was measured to determine the optimal inoculation amount. The optimum temperatures were determined by culturing at 28°C, 33°C, 37°C, 42°C, and 47°C. The initial pH of the skim milk medium was 5.0, 5.5, 6.0, 6.5, and 7.0, and the optimum initial pH was determined after cultivation.

#### 2.4.2 Effect of oxygen on strain growth

This test was carried out in two fermentors. The strain was inoculated into skim milk medium under optimal culture conditions. The pH of the fermentation was controlled at 5.8, the agitation speed was 85 rpm, and the culture was incubated at 37°C for 24 hr. Among them, the No. 1 fermenter was cultured under the above‐mentioned conditions, and the No. 2 fermenter was an aerated culture. Samples were taken every 2 hr during the fermentation, and the viable count and absorbance values (540 nm) were measured.

#### 2.4.3 Determination of optimal sugar concentration

The lactose concentrations in skim milk medium were controlled at 4.5%, 9.0% and 12%. Fermentation control parameters were as follows: pH 5.8, a stirring speed of 85 rpm, and a temperature of 37°C. The viable count and absorbance were measured every 2 hr during the fermentation.

#### 2.4.4 Determination of the best culture method

Batch culture, fed‐batch culture, and continuous culture are three commonly used culture methods in the fermentation industry. To establish a high‐density culture system, the three culture methods were compared. The medium used was skim milk medium, and the lactose concentration was adjusted to 9%.

### 2.5 Statistics

All experiments and analyses were carried out in triplicate and data represent the mean values. Microsoft Office Excel 2010 and SPSS (17.0) software were used for data analysis. One‐way ANOVA and independent‐sample *t* test were used for statistical analysis.

## 3 RESULTS AND DISCUSSION

### 3.1 Effect of lactose on the fermentation of *Lactobacillus helveticus*

**FIGURE 1:**
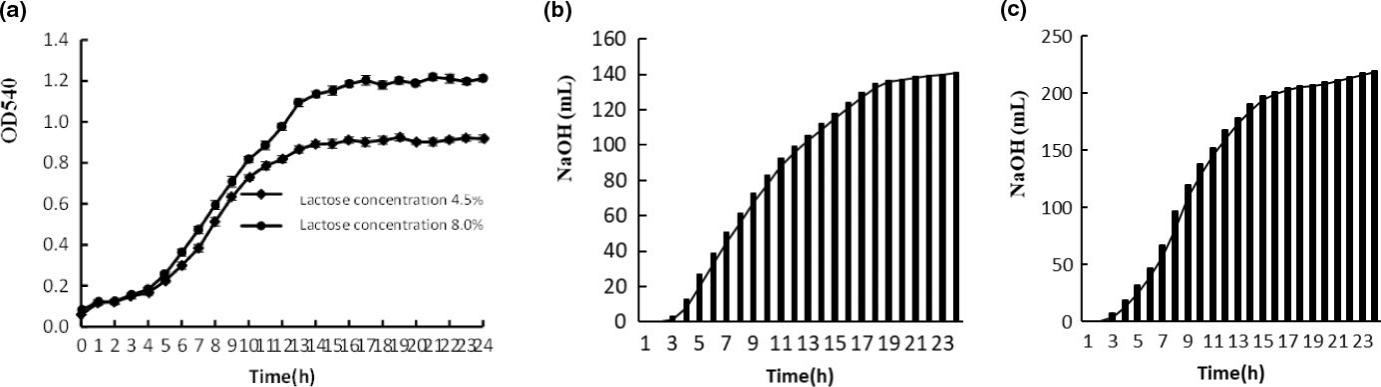
a was the growth curves in the medium contained different level of lactose, b represent the consumption of NaOH cultured in the media contained 4.5% lactose, c was the consumption of NaOH cultured in the media contained 8.0% lactose

The growth of *L. helveticus* CICC 22171 in different media and NaOH consumption during fermentation are shown in Figure 1. When the lactose concentration was 4.5%, the strain entered the logarithmic phase after 4 hr of fermentation, and began to enter the stationary phase after 11 hr. The absorbance of the cell density was 0.89, and the viable count was 8.3 × 10^8^ CFU/ml. When the lactose concentration was increased to 8.0%, the strain entered the stationary phase after 13 hr of fermentation. The absorbance of the cell density reached 1.2, and the number of viable cells reached 1.14 × 10^9^ CFU/ml. Compared to the 4.5% lactose concentration, the logarithmic phase was prolonged by 2 hr, and the viable count was significantly increased (*p* < .01). When the lactose concentration was 4.5%, the total NaOH consumption was 141.1 ml. When the lactose concentration was 8.0%, the total NaOH consumption was 219.3 ml, which was significantly higher than the former (*p* < .01). In addition, when the lactose concentration was 8.0%, the NaOH consumption rate in the logarithmic phase was significantly higher than that in the 4.5%. Therefore, the growth rate and metabolism of the strain were higher when cultured in medium with higher sugar concentration.

When the strain was cultured in skim milk medium, the final density was increased by adding lactose. In a previous study, lactulose was added to skim milk media to stimulate the proliferation of *Lactobacillus*. The results indicated that lactulose improves the quality of the skim milk media. The viable count of *Lactobacillus acidophilus*, *Lactobacillus bulgaricus,* and *Bifidobacterium* increased significantly (Oliveira et al., 2011), which was similar to this study. Bai reported that adding 1% lactose, 1% beef extract, and 3% corn pulp in MRS medium promotes the proliferation of LAB (Bai et al., 2007). Wu added 1.0% lactose, 1.0% tomato juice, 0.5% yeast extract, and 1.0% peptone to promote the high‐density proliferation of *Streptococcus thermophilus* and *Lactobacillus bulgaricus* (Wu, 2006). This indicated that carbon source regulation promoted the growth of *Lactobacillus*, thereby increasing the cell density.

### 3.2 Mechanism of the effect of lactose on the metabolism and proliferation of strain CICC 22171

#### 3.2.1 Effects of lactose on enzyme activity and gene expression

**FIGURE 2:**
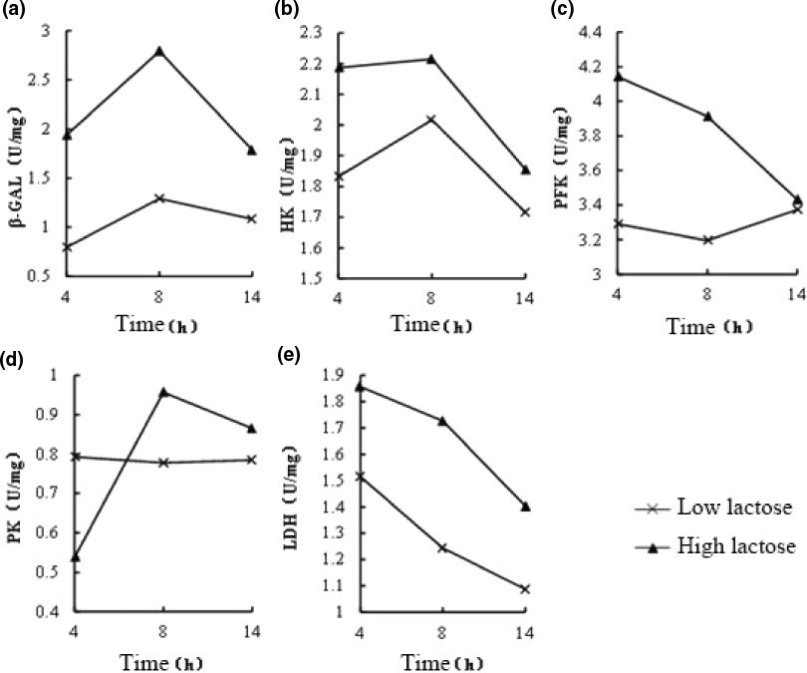
Effect of different concentration of lactose on key enzymes activity. a represent the effect of lactose concentration onβ‐GAL enzyme activity, b was the effect of lactose concentration on HK enzyme activity, c was the effect of lactose concentration on PFK enzyme activity, d represent the effect of lactose concentration on PK enzyme activity, e was the effect of lactose concentration on LDH enzyme activity

Different concentrations of lactose had a large influence on key enzyme activity in the fermentation process (Figure 2). Overall, the specific activity of intracellular enzymes in the high lactose medium was higher than that in the low lactose medium. It was most pronounced at 8 hr of fermentation. As shown in Figure 2, the specific activity of β‐GAL was significantly higher than that when cultured in low glucose (*p* < .01). The activities of HK and PFK were 10% and 22.4% higher than when cultured in low glucose; thus, the metabolic flux of the EMP pathway significantly increased.

*Lactobacillus helveticus* is a homotypic lactic acid fermenter, and β‐GAL, HK, PFK, PK, and LDH play an important role in the conversion of lactose into lactic acid. In this study, LDH activity showed a downward trend. In the early stage of fermentation, LDH activity was higher, and then began to decline, probably because the enzyme activity of LDH was affected by the accumulation of lactic acid (Qiu et al., 2007). The growth of the cells was the result of the comprehensive regulation of many metabolic pathways and various enzymes. By monitoring the activity of these five enzymes, the metabolic level of LAB could be reflected to a certain extent.

**FIGURE 3:**
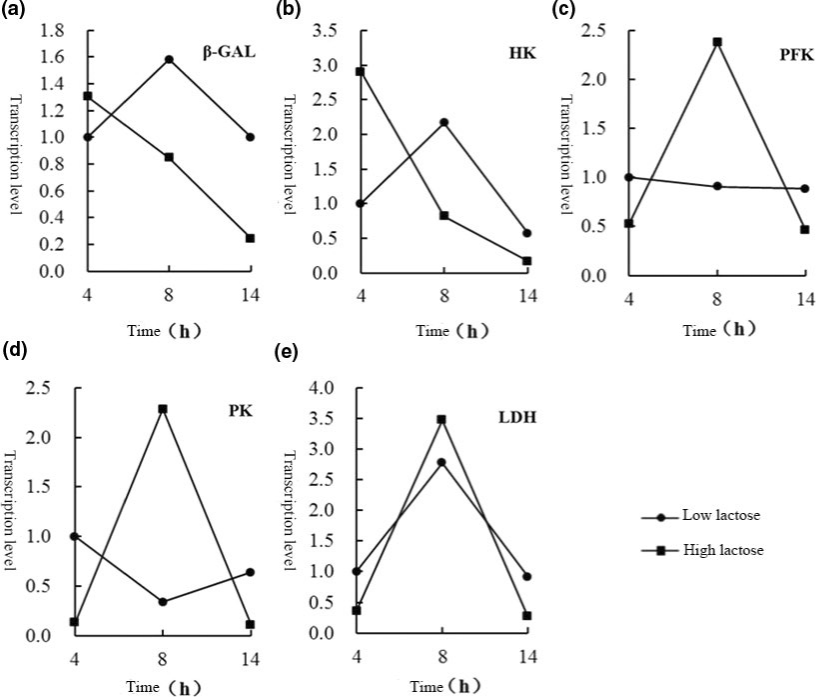
Effect of different concentration of lactose on the expression of genes. a was the change in the expression level of β‐GAL, b represent the expression level of HK, c was the change in the expression level of PFK, d represent the expression level of PK, e represent the changes in the expression level of LDH

In low‐sugar medium, the expression levels of β‐GAL and HK genes first increased and then decreased. There was no change in PFK gene expression. PK gene expression decreased and then increased. LDH gene expression increased and then was restored to normal levels. In high‐sugar medium, the expression levels of β‐GAL and HK genes gradually decreased. The expression of PFK and LDH genes increased and then decreased. PK gene expression increased sharply and then decreased sharply (Figure 3).

In the above results, the expression levels of PK and LDH genes at eight hours were several times those at four hours under high‐glucose concentration conditions. PK is a key enzyme that catalyzes the production of pyruvic acid by phosphoenolpyruvate (PEP), and LDH catalyzes the further synthesis of lactic acid by pyruvate. In addition to producing lactic acid, pyruvate is a synthetic precursor of other materials (Viana et al., 2005). Fermentation after eight hours was the middle stage of the logarithmic growth phase, which is a period in which the cells proliferate in a large amount. The data showed that high‐sugar stimulated the proliferation of the strain.

#### 3.2.2 Effect of lactose on strain protein expression

After two‐dimensional electrophoresis, Image Master 2D Platinum software was used for analysis. In sample L (low‐sugar media), 1,409 protein spots were found at pH 4–7 and 1,348 protein spots were isolated from sample H (high‐sugar media). There were six differential protein spots, of which three were up‐regulated (up >2 fold) and three down‐regulated. At pH 3–10, seven differential protein spots were detected, of which four were up‐regulated and three down‐regulated. A total of 13 differentially expressed proteins were obtained, 11 of which were identified (Table 1). The up‐regulated list included phosphofructokinase, cysteine synthase, glyceraldehyde‐3‐phosphate dehydrogenase, amino acid transporter, triose phosphate isomerase, adenosine triphosphate binding cassette transporter, and phenylalanine‐tRNA synthetic enzyme.

**TABLE 1:** Sequence similarity comparison with previously reported proteins

Protein point	The matching protein	Matched peptide of protein sources
B408	6‐Phosphofructokinase	*L. acidophilus* NCFM
B368	Cysteine synthase	*L. helveticus* DPC 4571
B466	Glyceraldehyde−3‐phosphate dehydrogenase	*L. helveticus* DPC 4571
B470	Mannose‐specific PTS system component IIAB	*L. helveticus* DPC 4571
B130	PTS enzyme II, ABC component	*L. helveticus* DPC 4571
B257	Uracil phosphoribosyltransferase	*L. helveticus* H10
B352	Methylenetetrahydrofolate dehydrogenase	*L. helveticus* DPC 4571
B198	Amino acid transporter	*L. helveticus* DPC 4571
B204	Triosephosphate isomerase	*L. helveticus* DPC 4571
B208	Adenosine triphosphate binding box transporter	*L. helveticus* CNRZ32
B220	Phenylalanine‐tRNA synthase	*L. helveticus* CNRZ32

The proteomic approach efficiently identified differentially expressed proteins induced by the change in the carbon source (McLeod, 2010). After analysis, these identified differentially expressed proteins were mainly involved in processes such as sugar metabolism, amino acid synthesis, nucleotide synthesis, and substance transport. Among them, phosphofructokinase is an important control point in the EMP pathway. The rate of glycolysis depends primarily on phosphofructokinase. These data indicated that when the strain was growing in high‐sugar medium, sugar metabolism was obviously active.

#### 3.2.3 Transcriptomic analysis

According to the GO enrichment analysis, 885 genes were up‐regulated and 766 genes were down‐regulated. In the cellular component (CC) group, 18 genes were up‐regulated and 0 genes were down‐regulated. In the molecular function (MF) group, 39 genes were up‐regulated and 30 genes were down‐regulated. In the biological process (BP) group, 76 genes were up‐regulated and 42 genes were down‐regulated. Regarding the CC annotation, the up‐regulated genes were located in the outer region of the cell, and mainly involved S‐layer proteins, phosphoric acid, calcium transport ATPase in the cell membrane and other genes. The up‐regulated genes annotated in the MF group mainly included material transmembrane transport hydrolase activity, amino acid transport activity, ATPase activity coupled with transmembrane transport, and calcium transport ATPase activity. Down‐regulated genes mainly had carbohydrate binding effects, nucleic acid binding effects, DNA‐methyltransferase activity, and n‐methyltransferase activity. In the BP annotation, a series of processes including fatty acid metabolism, fatty acid biosynthesis, ATP biosynthesis, amino acid biosynthesis, ion transport, and amino acid transport were up‐regulated. In contrast, the down‐regulated genes were mainly involved in DNA methylation and DNA restrictive modification processes (Table 2). According to the analysis of COG types, 244 unigenes were classified and annotated in COG, involving 18 different functions. Of the entire COG functional categories, one of the biggest proportions involved predicting a global function, followed by transport and metabolism of amino acids and transport and metabolism of inorganic ions. KEGG pathway enrichment analysis revealed that four metabolic pathways were up‐regulated and three were down‐regulated, according to the screening criteria (Table 3). The up‐regulated pathways increased the synthesis of fatty acids and metabolism of biotin, while the down‐regulated pathways were related to phosphotransferase activity and gluconeogenesis.

**TABLE 2:** Annotation of differential genes in the three classifications of GO

	GO class number	Number of genes	GO annotation
Up‐regulated gene in CC	GO:0005576	18	Extracellular region
Up‐regulated gene in MF	GO:0016820	10	Hydrolase activity, catalyzing transmembrane movement of substances
	GO:0015171	9	Amino acid transmembrane transporter activity
	GO:0015662	10	ATPase activity, coupled to transmembrane movement of ions, phosphorylative mechanism
	GO:0015444	4	Magnesium‐importing ATPase activity
	GO:0005388	5	Calcium‐transporting ATPase activity
Down‐regulated genes in MF	GO:0008170	5	N‐methyltransferase activity
	GO:0009007	4	DNA‐methyltransferase activity
	GO:0030246	4	Carbohydrate binding
	GO:0003676	13	Nucleic acid binding
Up‐regulated genes in BP	GO:0006633	7	Fatty acid biosynthetic process
	GO:0006754	9	ATP biosynthetic process
	GO:0006631	6	Fatty acid metabolic process
	GO:0003333	9	Amino acid transmembrane transport
	GO:0006812	11	Cation transport
	GO:0015693	4	Magnesium ion transport
	GO:0006629	10	Lipid metabolic process
	GO:0006811	11	Ion transport
	GO:0009085	4	Lysine biosynthetic process
	GO:0009089	4	Lysine biosynthetic process via diaminopimelate
Down‐regulated genes in BP	GO:0006306	7	DNA methylation
	GO:0009307	16	DNA restriction–modification system
	GO:0032775	8	DNA methylation on adenine
	GO:0006304	10	DNA modification

**TABLE 3:** Significant enrichment pathways

Pathway ID	log2FC	*p*‐value	Pathway description
Raised pathways			
map00061	2.97	3.45E‐04	Fatty acid biosynthesis
map00780	2.75	2.64E‐02	Biotin metabolism
map00300	2.16	2.66E‐02	Lysine biosynthesis
map02020	1.84	4.57E‐02	Two‐component system
Cut pathways			
map02060	1.64	4.31E‐03	Phosphotransferase system (PTS)
map00010	1.64	1.01E‐02	Gluconeogenesis
map00520	1.64	2.45E‐02	Amino sugar and nucleotide sugar metabolism

To grow and reproduce, *L. helveticus* must ingest nutrients from the medium. The substances that enter the cell must undergo transmembrane transport. Very few of these substances can enter and exit the cell directly through the cell membrane. Transmembrane transport of most nutrients is associated with various protein carriers on the membrane. Most of these proteins have hydrolase activity (Sadat‐Mekmene et al., 2011; Yamamoto et al., 1996). In this study, after the regulation of the carbon source and the increased lactose concentration, the expression of related enzymes in transmembrane transport of substances was significantly improved. This indicated that the utilization of nutrients was accelerated, and the growth rate was increased, which was more conducive to the proliferation of *L. helveticus*. In addition, the expression level of magnesium (Mg^2+^) transport‐related genes was also significantly up‐regulated. Mg^2+^ is a cofactor or activator of many enzymes in the cell, including almost all phosphorylation processes, such as glycolysis. The regulation of enzyme activity by Mg^2+^ is usually achieved by direct binding of the substrate to the active site or by maintaining the active conformation of the enzyme (Cowan, 2002). Some studies reported that Mg^2+^ also plays an important role in transcription and translation (Cong et al., 2012; Smith et al., 1993). In this study, Mg^2+^ transport‐related genes were highly expressed; the activity of the related metabolic pathway enzyme was activated, the overall metabolic level of the cell was increased, and cell proliferation was accelerated. Other biological processes, such as amino acid transport and synthesis, lipid metabolism, and other related genes were also up‐regulated, further confirming that high‐glucose conditions could stimulate rapid proliferation of LAB. Accordingly, analysis of down‐regulated genes revealed that the expression level of the DNA methylase gene was down‐regulated. DNA methylation is one of the first modification pathways discovered. Numerous studies have shown that DNA methylation can cause changes in DNA conformation, DNA stability, and DNA‐protein interactions, thereby inhibiting gene expression (Hubácek, 1992). In this study, the expression of the DNA methylase gene was down‐regulated, indicating that inhibition of gene expression was abolished and DNA expression levels were increased, reflecting the fact that the growth and metabolism of the strain were more active under high‐glucose conditions.

#### 3.2.4 Comprehensive analysis

According to the analysis of the detection of key enzymes, proteomics and transcriptomics, the up‐regulated enzymes (genes) included pyruvate kinase, acetyl‐CoA carboxylase, phenylalanine‐tRNA synthase, amino acid permeable enzyme, and uracil phosphoribose transferase. (Table 4 **)**. These enzymes were mainly involved in sugar metabolism, fatty acid synthesis, amino acid synthesis, nucleotide synthesis, material transport, energy metabolism, and other processes. In conclusion, when *L. helveticus* was cultured in high‐glucose medium, metabolism in general was increased, which was conducive to the rapid growth and reproduction of the strain.

**TABLE 4:** The up expressed enzymes or genes

Metabolic process	Related enzymes (genes)
Glycometabolism	Hexokinase, 6‐Phosphofructokinase, Pyruvate kinase, Lactate Dehydrogenase, Enolase,
	Glyceraldehyde‐3‐phosphate dehydrogenase, Lactose permease, β‐galactosidase, Triosephosphate isomerase
Fatty acid synthesis	3‐Oxyacyl Carrier Protein Synthase, 3‐Oxyacyl Carrier Protein Reductase, Acyl carrier protein dehydrogenase,
	Malonic acid monoacyl CoA acyl carrier protein, Enyl carrier protein reductase, Acetyl‐CoA carboxylase
Amino acid synthesis	Phenylalanine‐tRNA synthase, Cysteine synthase, Lysine synthesis‐related enzymes
Material transport	Amino acid transporter, Adenosine triphosphate binding box transporter, Amino acid permeable enzyme,
	Calcium transport atpase, Magnesium transport atpase, H(+)/Cl(−) Exchange Transporter, Na(+)/H(+) Exchange
Metabolism of nucleic acid	Uracil phosphoribosyltransferase

### 3.3 Establishment of a high‐density culture system

#### 3.3.1 The inoculum size, temperature, and initial pH value were determined

**FIGURE 4:**
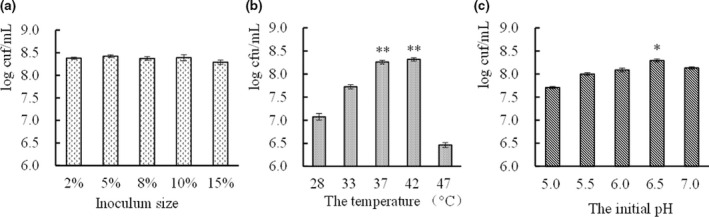
The effect of culture conditions on the growth of strain, a was the effect of inoculum size on the growth of strain, b represent the temperature, c represent the effect of initial pH

According to Figure 4a, the viable count of strain CICC 22171 is the highest when the inoculation quantity is 5%. However, there was no statistical difference (*p* > .05). Therefore, 5% inoculation amount was selected. At 37°C and 42°C, the viable counts of *L. helveticus* were significantly higher than when cultured at other temperatures (*p* < .01). However, there was no significant difference between 37°C and 42°C (*p* > .05). From the perspective of energy consumption, 37°C was selected as the culture temperature of the strain (Figure 4b). When the initial pH value was 6.5, the viable counts were significantly higher than under other pH conditions (*p* < .05). Therefore, 6.5 was chosen to be the initial pH value (Figure 4c).

The inoculation amount was related to the growth rate of the strain in the fermentation tank. Excessive inoculation will cause insufficient dissolved oxygen and affect product synthesis. Too small of an inoculation will lengthen the cultivation time and reduce the productivity of the fermentation equipment. A large number of enzymes are involved in bacterial metabolism and enzyme activity is correlated with temperature. Therefore, selecting an appropriate culture temperature was beneficial to the rapid proliferation of *L. helveticus*. In addition, the pH value could affect the absorption of nutrients by *L. helveticus*. Thus, at the optimal temperature and in the optimal pH range, the cells had a fast growth rate and a strong metabolism.

#### 3.3.2 The effect of oxygen on the growth of *L. helveticus*

**FIGURE 5:**
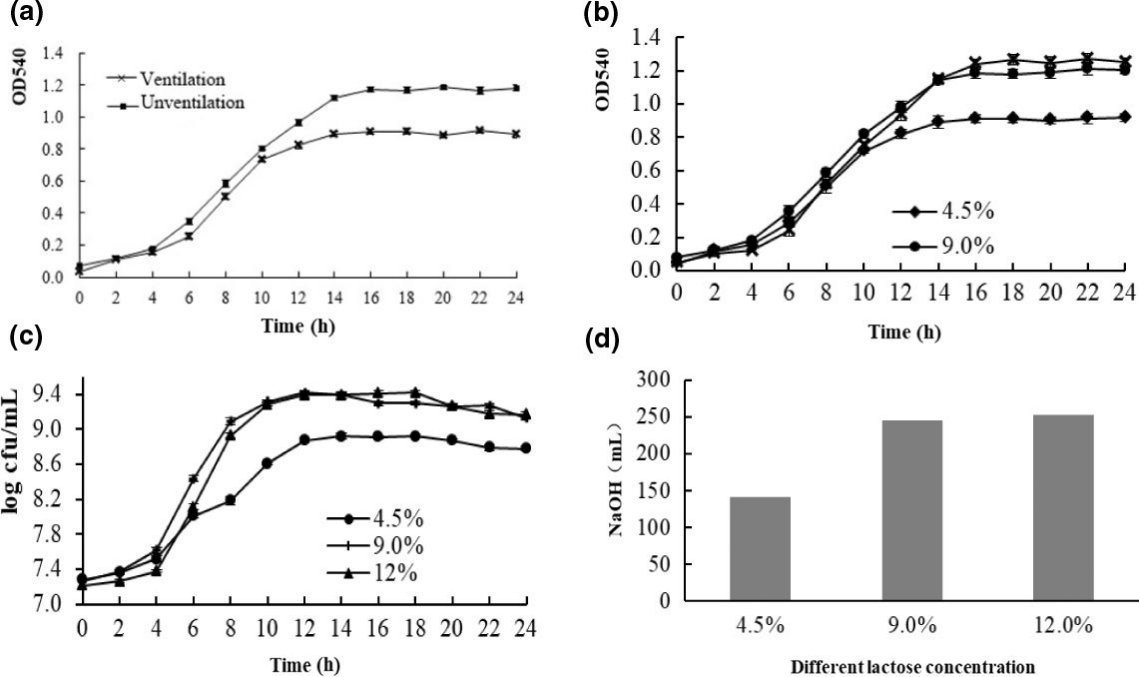
a was the effect of O_2_ on the growth of *L. helveticus*, b represent the growth curve of the strain in three media, c represent viable count curve of the strain in three media, d was the consumption of NaOH of the strain in three media

As shown in Figure 5a under the two culture conditions, after 4 hr the bacterial strains entered the logarithmic phase, and after 10 hr the aerated bacterial strains entered the stationary phase. In contrast, the logarithmic phase could be extended to 14 hr in the anaerobic culture, and the corresponding bacterial density was significantly higher than that in the aerated culture (*p* < .01).

Lactic acid bacteria is an anaerobic or facultative anaerobic microbe, and the oxygen tolerance varies between strains. Some strains have a good tolerance of oxygen and some strains do not. Under the conditions of high dissolved oxygen training, not only can the bacteria produce more metabolic by‐products such as acetic acid, formic acid and ethanol, the growth rate of the strain, utilization of nutrients, density of the strain, and viable count are reduced (Niel et al., 2002). It has been reported that *Lactobacillus* can tolerate a stirring rate range of 50–100 rpm (Li et al., 2009). In this study, the stirring rate was 85 rpm, which had no negative effect on the growth of the strain. However, under aerated culture conditions, the density of the strain significantly decreased and growth metabolism was retarded. Aeration increased the dissolved oxygen in the medium, which was unfavorable to the growth of the strain, demonstrating that the strain CICC 22171 was sensitive to oxygen. Thus, to realize a high‐density culture, aeration should be strictly controlled.

#### 3.3.3 Determination of the optimal lactose concentration

The growth of the strains in the three media is compared in Figure 5. The results showed that the growth of the strain in the high‐sugar medium (9.0% and 12.0%) was significantly better than that in the low‐sugar medium (4.5%). The viable count was about three times that in the low‐sugar medium, and the total NaOH consumption was also significantly higher than that in the low‐sugar medium.

There was no statistical difference in the viable count at the end (*p* > .05) for strains grown in the two kinds of sugar media, but the growth curve showed a slightly different growth trend. When cultured in a culture medium containing 9.0% lactose, the time it took for the strain to enter the logarithmic phase was four hours, and the time to enter the stationary phase was 14 hr. However, when cultured in culture medium containing 12.0% lactose, the time it took for the strain to enter the logarithmic and stationary phases was relatively delayed by 1–2 hr. It may be that since the nutrient concentration was higher, a higher osmotic pressure was generated, affecting the growth and metabolism of the strain. Therefore, considering the fermentation cycle, energy consumption and material consumption, the culture medium containing 9.0% lactose was selected for subsequent experiments.

#### 3.3.4 Determination of the best cultivation mode

The final obtained bacterial density, viable cells, and residual sugar concentration were used as indicators to compare the three culture methods. As shown in Table 5, the fed‐batch culture is the best culture method. The maximum viable cells reached 3.98 × 10^9^ CFU/ml.

**TABLE 5:** Comparison of the three different cultures

Way of culture	OD value	Number of living bacterium (cfu/mL)	The final sugar concentration (g/L)
Batch cultivation	1.183	1.85 × 109	8.1
Fed‐batch culture	1.238	3.98 × 109	5.6
Continuous culture	0.646	2.12 × 109	22.6

A direct‐investment starter is the focus of dairy industry research. In the production of a starter culture, the most important problem is the acquisition of high‐density cell culture. Therefore, the strain must be cultured in high density. At present, studies on the high‐density culture of LAB mainly focus on the optimization of medium and the application of buffer salts and chemical neutralization to control pH (Wang et al., 2009). Using alkali neutralization, the effect of different culture methods on the high‐density proliferation of the strain was studied. The results showed that the viable count of the fed‐batch culture method was 2.15 times and 1.88 times higher than that of batch and continuous culture, respectively.

## 4 CONCLUSION

Increasing the lactose concentration in skim milk medium could promote the growth and reproduction of *L. helveticus*, thereby increasing the cell density in the medium. Analysis of changes in key enzyme activities and gene expression levels showed that the high‐sugar medium promoted lactose transport and a quick start of sugar metabolism. In addition, proteomics and transcriptomics analysis showed that *L. helveticus* had increased metabolism overall when fermented in a high‐sugar medium, which was beneficial to the rapid proliferation of bacterial cells. In summary, increasing lactose concentration promoted the proliferation of *L. helveticus*. The intrinsic relationship between *L. helveticus* and related metabolic pathways was clarified, which provided theoretical guidance for subsequent high‐density fermentation regulation.

## Data Availability

All data, models, and code generated or used during the study appear in the submitted article.
